# Phylodynamic reconstruction of the spatiotemporal transmission and demographic history of coxsackievirus B2

**DOI:** 10.1186/s12859-015-0738-2

**Published:** 2015-09-21

**Authors:** Hui-Wen Huang, Yao-Shen Chen, Jeff Yi-Fu Chen, Po-Liang Lu, Yung-Cheng Lin, Bao-Chen Chen, Li-Chiu Chou, Chu-Feng Wang, Hui-Ju Su, Yi-Chien Huang, Yong-Ying Shi, Hsiu-Lin Chen, Bintou Sanno-Duanda, Tsi-Shu Huang, Kuei-Hsiang Lin, Yu-Chang Tyan, Pei-Yu Chu

**Affiliations:** Department of Anesthesiology, Kaohsiung Chang Gung Memorial Hospital and Chang Gung University College of Medicine, Kaohsiung, ROC Taiwan; Department of Biological Sciences, National Sun Yat-Sen University, Kaohsiung, ROC Taiwan; Division of Infectious Diseases, Kaohsiung Veterans General Hospital, Kaohsiung, ROC Taiwan; Division of Microbiology, Department of Pathology and Laboratory Medicine, Kaohsiung Veterans General Hospital, Kaohsiung, ROC Taiwan; Department of Internal Medicine, National Yang-Ming Medical University, Taipei, ROC Taiwan; Department of Biotechnology, Kaohsiung Medical University, Kaohsiung, ROC Taiwan; School of Medicine, College of Medicine, Kaohsiung Medical University, Kaohsiung, ROC Taiwan; Department of Laboratory Medicine, Kaohsiung Medical University Hospital, Kaohsiung, ROC Taiwan; Institute of Bioscience and Biotechnology, National Taiwan Ocean University, Keelung, ROC Taiwan; Department of Medical Laboratory Science and Biotechnology, College of Health Sciences, Kaohsiung Medical University, Kaohsiung, ROC Taiwan; Department of Pediatrics, Kaohsiung Medical University Hospital, Kaohsiung, ROC Taiwan; Department of Respiratory Therapy, College of Medicine, Kaohsiung Medical University, Kaohsiung, ROC Taiwan; Department of laboratory medicine, Edward Francis Small Teaching Hospital, Banjul, Gambia; Department of Medical Imaging and Radiological Sciences, Kaohsiung Medical University, Kaohsiung, Taiwan; Center for Infectious Disease and Cancer Research, Kaohsiung Medical University, Kaohsiung, Taiwan

**Keywords:** Coxsackievirus B2, Phylodynamic, Spatiotemporal transmission, Demographic history, Molecular epidemiology

## Abstract

**Background:**

Studies regarding coxsackievirus (CV) tend to focus on epidemic outbreaks, an imbalanced topology is considered to be an indication of acute infection with partial cross-immunity. In enteroviruses, a clear understanding of the characteristics of tree topology, transmission, and its demographic dynamics in viral succession and circulation are essential for identifying prevalence trends in endemic pathogens such as coxsackievirus B2 (CV-B2). This study applied a novel Bayesian evolutionary approach to elucidate the phylodynamic characteristics of CV-B2. A dataset containing 51 VP1 sequences and a dataset containing 34 partial 3D^pol^ sequencing were analyzed, where each dataset included Taiwan sequences isolated during 1988–2013.

**Results:**

Four and five genotypes were determined based on the 846-nucleotide VP1 and 441-nucleotide 3D^pol^ (6641–7087) regions, respectively, with spatiotemporally structured topologies in both trees. Some strains with tree discordance indicated the occurrence of recombination in the region between the VP1 and 3D^pol^ genes. The similarities of VP1 and 3D^pol^ gene were 80.0 %–96.8 % and 74.7 %–91.9 %, respectively. Analyses of population dynamics using VP1 dataset indicated that the endemic CV-B2 has a small effective population size. The balance indices, high similarity, and low evolutionary rate in the VP1 region indicated mild herd immunity selection in the major capsid region.

**Conclusions:**

Phylodynamic analysis can reveal demographic trends and herd immunity in endemic pathogens.

**Electronic supplementary material:**

The online version of this article (doi:10.1186/s12859-015-0738-2) contains supplementary material, which is available to authorized users.

## Background

Coxsackievirus (CV) B2 belongs to the species *Enterovirus* B (EV-B) in the genus *Enterovirus*, family *Picornaviridae*. Long-term surveillance of circulating EV serotypes has identified three main outbreak patterns: epidemic, endemic and sporadic [1]. However, most studies of EV have focused on ‘epidemic’ pathogens rather than on ‘sporadic’ or ‘endemic’ pathogens such as CV-B2; thus, no phylogenetic analysis of CV-B2 has been reported. In terms of epidemiology, a virus is considered ‘endemic’ if its population remains constant within a population in a geographic area; it is considered ‘sporadic’ if it appears often but not regularly. The circulation model of individual serotypes differs by season, geographic location, herd immunity and viral genotypes [1, 2]. Thus, analysis history of spatiotemporal transmission and viral population dynamics can reveal trends in their patterns of circulation and succession.

In Taiwan, episodes of unusually high CV-B2 activity were detected in 1999, 2002, 2003 and 2006, and infections are still reported almost annually to the Taiwan Centers for Disease Control (Additional file 1). CV-B2 has an endemic circulation pattern worldwide where it comprises 1.5 %–6.0 % of the annual reported EV rates (Additional file 2) [1, 3–7]. The peak activity of CV-B2 (>10 %) is rare, but it is commonly identified as one of the top 15 most frequently detected EV serotypes globally [3, 4]. CV-B2 shares many common properties and clinical features with other CV-Bs but they have different outbreak tempos, and overwhelming infection may develop in infants (<1 year) [8]. CV-B infection is also associated with myocarditis, polymyositis and chronic autoimmune diseases such as insulin-dependent diabetes mellitus [9]. Moreover, outbreaks of emerging and re-emerging infectious diseases are strongly associated with climate change and human activity [10]. Increased CV-B1 activity levels and severe infections of young infants have been reported in the USA (2007–2008) and South Korea (2008–2009) [11, 12]. Therefore, identifying the phylodynamics of CV-B2 is essential for bridging relationships between successive prevalent serotypes and identifying EV circulation trends.

The Bayesian Evolutionary Analysis Sampling Trees (BEAST) program combined various models to infer complex relationships among viral phylodynamics [13]. In addition to genetic variation, viral spatial and temporal components are fundamental issues that require clarification as the dispersal pattern expands. A clear understanding of the tree topology, transmission history, and demographic dynamics of an endemic pathogen such as CV-B2 during viral succession and circulation is needed to identify its prevalence trends. In EVs, the VP1 gene sequence encodes the major serotypic epitope, thereby making it the target choice for molecular typing [14, 15]. Recombination, which is reportedly a common phenomenon in the EV family, can be identified based on phylogenetic incongruencies in the VP1 and 3D^p^°^l^ gene regions [16–18] because 3D^pol^ is located in the last codon region of the EV genome. To explore the phylogenetic structure of endemic pathogens such as CV-B2, both VP1 and 3D^pol^ regions were ananlyzed by the BEAST program to elucidate its phylodynamic properties and reconstruct its spatiotemporal transmission history.

## Methods

### Ethics statement

Twenty virus strains were randomly selected from CV-B2-positive viral stock. These virus strains were collected during 1988–2013 by the Taiwan Centers for Disease Control, Taiwan (CDC-TW) and two medical centers in Southern Taiwan, Kaohsiung Veterans General Hospital and Kaohsiung Medical University Hospital. This study was approved by the ethics committees of both hospitals. All samples were de-identified and analyzed anonymously. Informed consent was waived because we only conducted experiments on viral isolates obtained from clinically necessary laboratory procedure and no harm to the patients was anticipated because none of the patients’ medical history was studied.

### Specimens, viral RNA extraction, reverse transcription-polymerase chain reaction (RT-PCR) and sequencing

Among the twenty CV-B2 strains, fifteen were isolated from throat swabs and five were isolated from rectal swabs or stool. Forty percent of the infected patients were infant younger than 1 year (mean age: 2 years; range: 1 month − 11 years). The male-female ratio was 1.86:1. Viral RNA was extracted using a QIAmp viral RNA purification kit according to the manufacturer instructions (Qiagen, Chatsworth, CA, USA). The RT-PCR and sequencing were performed as described previously [19, 20]. Table 1 lists the degenerate primer pairs used for amplifying and sequencing the VP1 and partial 3D^pol^ genes. The purified PCR products were sequenced with an ABI Prism Ready Reaction Dideoxy Terminator cycle sequencing kit (Model 3730, Version3.4, Applied Biosystems, Foster City, CA, USA). The obtained sequence data were submitted to GenBank under accession number*s* AB862097, AB862101, AB862107, AB862115–AB862116, LC055763–LC055766, LC055768–LC055778, and LC057297–LC057316.

**Table 1:** Primer sets used for amplification and sequencing of Coxsackievirus B2

Gene	Primer^a^	Sequence	Position^b^	Reference
VP3	2400F	GCTTTGTGTCTGCMTGYAATGA	2372-2393	CDC-TW^c^
VP1	222R	CICCIGGIGGIAYRWACAT	2909-2891	[20]
VP1	292F	MIGCIGYIGARACNGG	2550-2565	[20]
2A	011R	GCICCIGAYTGITGICCRAA	3327-3308	[20]
3D^pol^	PY-03F	GTYACMTATGTGAARGATG	6383-6398	This study
3D^pol^	PY-04R	CTTCATTGGCATTACTGGATG	7104-7085	This study

^a^F: Forward primer, R: reverse primer

^b^Numbering system used for the Coxsackievirus B2 strain (Accession No. AF085363)

^c^Primer designed by the Centers for Disease Control, Taiwan

### Model selection and sequence variation detection

Multiple sequence alignments were performed with T-coffee [21, 22]. In addition to the 20 CV-B2 strains obtained in this study, the sequences available in GenBank were also included. Sequences were excluded if they had a nonsense or frame-shift mutation pattern in a single strain. The final VP1 dataset comprised 51 sequences and the partial 3D^pol^ dataset comprised 34 sequences (Additional file 3).

The jModelTest v 2.1.4 program [23] was used to select the best-fit model for both datasets according to Akaike’s information criterion. A 4-category Generalised Time Reversible [24] model with a Gamma distribution (GTR + G) was used for VP1 and 3D^pol^ (G = 0.1570 and G = 0.1380, respectively). For the BEAST estimation, eight model compositions were compared: two substitution models (GTR + G and Shapiro-Rambaut-Drummond-2006 (SRD06) [25]), two population models [constant (CON) population size and Bayesian skyline plot (BSP)], and two relaxed molecular clock models [uncorrelated log-normal distribution and uncorrelated exponential distribution (uced)] [26]. The Bayes Markov chain Monte Carlo (BMCMC) analysis results were sampled regularly until convergence was reached.

Recombination was estimated using the Recombination Detection Program (RDP v3.44) with the default settings [27]. Pairwise comparisons of nucleotide and amino acid (aa) sequences were calculated based on *p*-distance matrices implemented in the Molecular Evolutionary Genetics Analysis version 6 (MEGA6) program. The ratio of non-synonymous/synonymous substitutions rate (d*N*/d*S*) was used as an indicator of the selection force acting on the coding sequences and was detected by using Single Likelihood Ancestor Counting (SLAC) method via the Datamonkey website [28].

### Phylodynamic analyses

Phylogenetic analyses were performed as described previously [19]. The BMCMC tree analyses were performed using BEAST v.1.8.2 [13]. Nodal reliability of BMCMC trees were estimated according to posterior probability (PP). The breakpoint for significant support was set to a PP value > 0.9. The BEAST program was used to co-estimate the nt substitution rate and population growth model in the Tracer v.1.6 program [29]. Parameters with effective sample size (ESS) values > 200 were considered reliable. Briefly, each run of VP1 and 3D^p^°^l^ had a chain length of 6 million generations, and sampling was performed once every 6000 generations. When the major parameters with ESS value >200 were considered as candidates for the best model composition, the best model for both the VP1 and 3D^pol^ regions was SRD06 + UCED + CON (Additional file 4). All estimation parameters were shown as mean and 95% highest posterior density (HPD). Demographic changes over time (Neτ) were also explored by BSP method [30]. The FigTree v.1.4.2 program was used to construct and visualize the maximum clade credibility tree. The SPREAD program [31] was used to estimate the route of virus transmission and to calculate the Bayes factors (BFs) associated with major virus dispersal routes. A major virus dispersal route was defined as a route in which at least two locations had BF values >3. Furthermore, the TreeStat program in the BEAST package was used to summarize statistical tests of neutrality (Fu & Li’s *D*) and to calculate three tree-balance statistical measures: cherry count (*Cn*), Colless’s tree imbalance (*Ic*), and *B*_*1*_ [32].

## Results and discussion

**Fig. 1:**
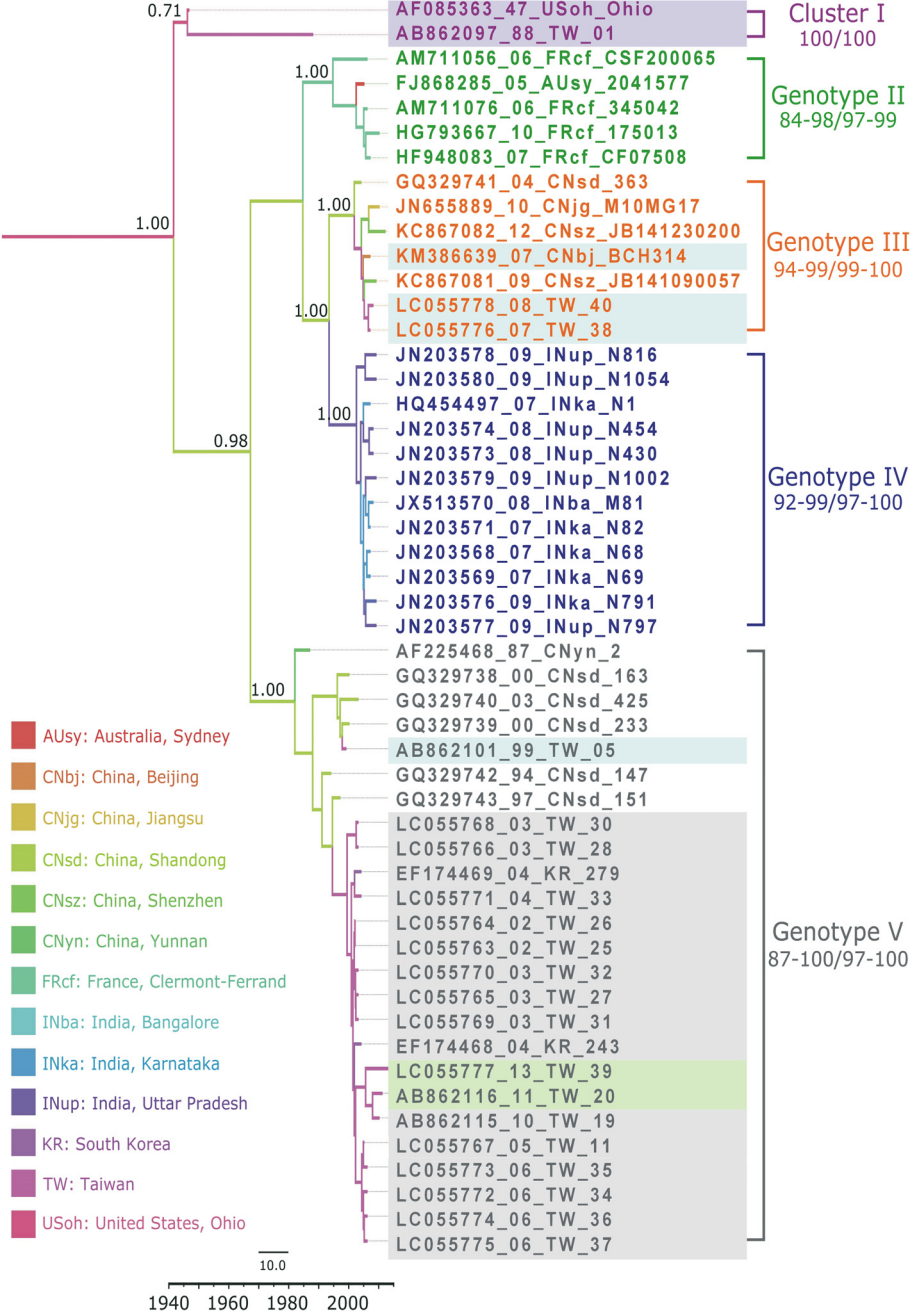
Maximum clade credibility (MCC) phylogeny of 51 VP1 sequences of coxsackievirus B2. For each branch, the thickness indicates the state probability, and the color indicates the most probable location. Support values are indicated on the major nodes. The genotypes and nucleotide/amino acid similarity within genotypes are shown on the right. For each strain name, the VP1 genotypes are differentiated by colors (Cluster I: purple, Genotype II: green, Genotype III: orange, Genotype IV: blue, and Genotype V: grey), whereas the 3D^pol^ genotypes are differentiated by shading (Genotype A: purple, Genotype B: green, Genotype C: orange, Genotype D: blue, and Genotype E: gray). The branch length is proportional to the evolutionary time, and the scale bar is proportional to calendar time

**Fig. 2:**
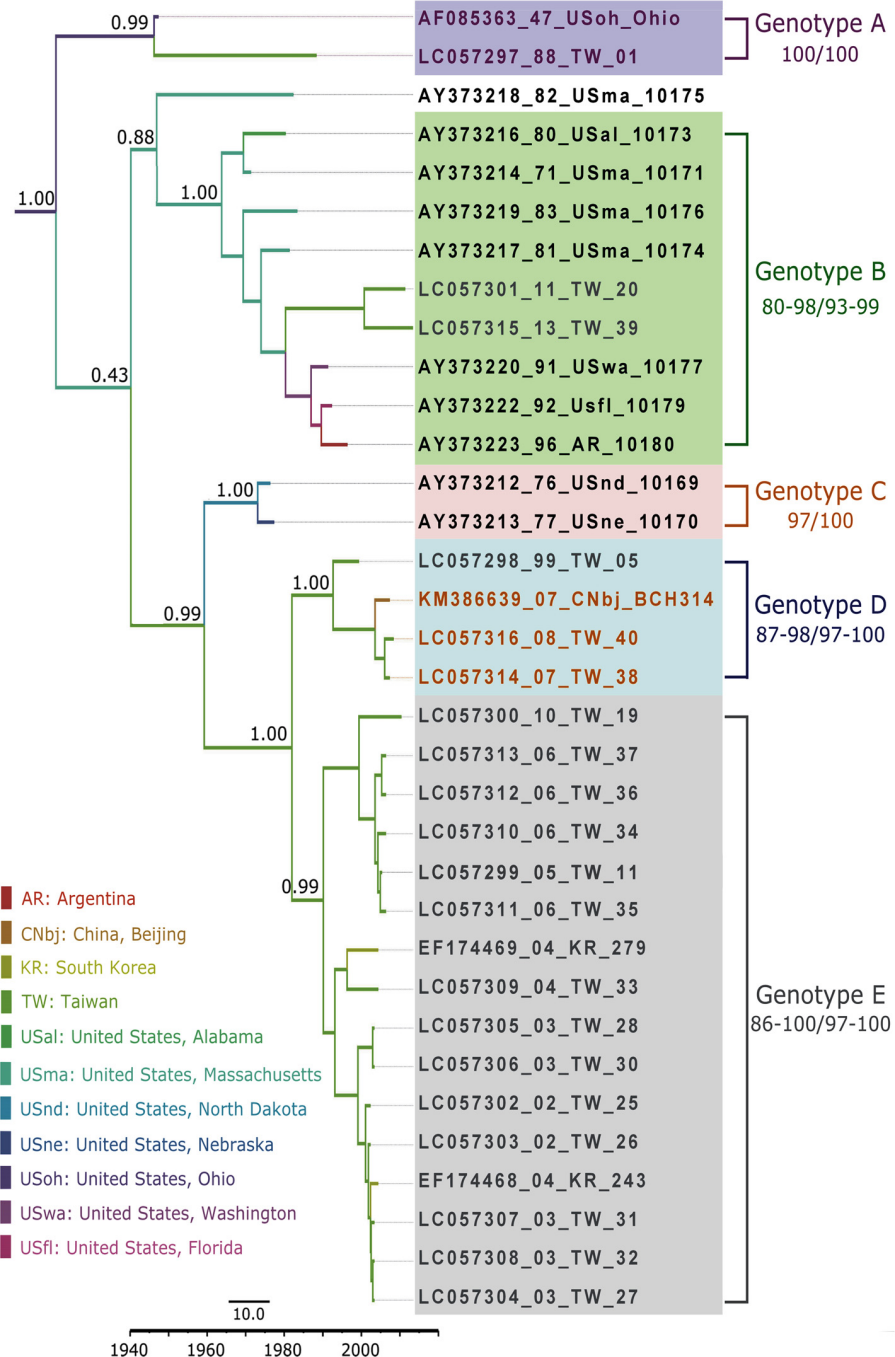
Maximum clade credibility (MCC) phylogeny of 34 3D^pol^ sequences coxsackievirus B2. For each branch, the thickness indicates the state probability and the color indicates the most probable location. Support values are indicated on the major nodes. The genotypes and nucleotide/amino acid similarity within genotypes are shown on the right. For each strain name, the VP1 genotypes are differentiated by colors (Cluster I: purple, Genotype II: green, Genotype III: orange, Genotype IV: blue, and Genotype V: grey), whereas the 3D^pol^ genotypes are differentiated by shading (Genotype A: purple, Genotype B: green, Genotype C: orange, Genotype D: blue, and Genotype E: gray). The branch length is proportional to the evolutionary time, and the scale bar is proportional to calendar time

**Fig. 3:**
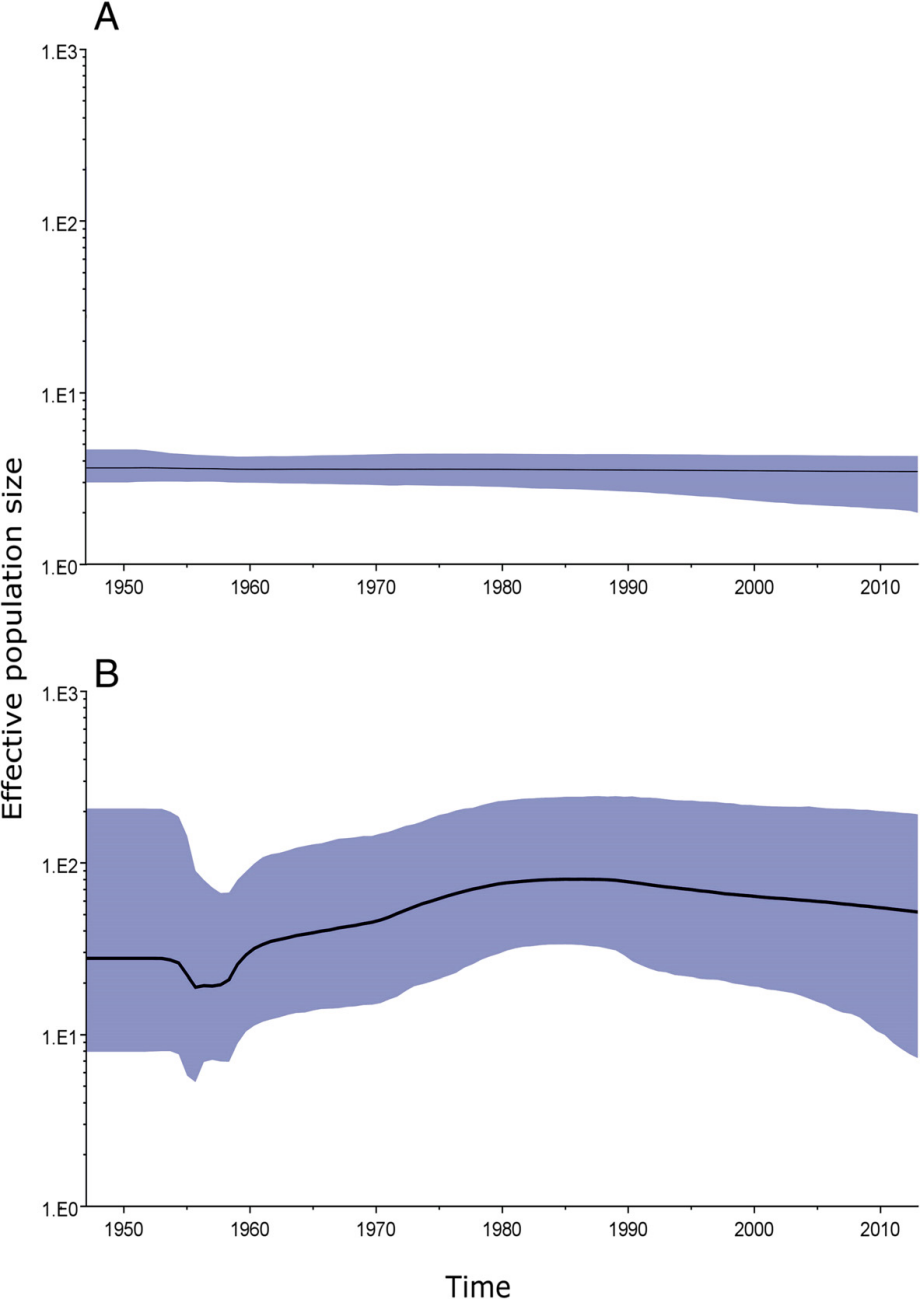
Bayesian skyline plot of (**a**) 51 VP1 sequences and (**b**) 34 3D^pol^ sequences. The x-axis is the time scale (years) and the y-axis is the logarithmic Neτ scale (Ne is the effective population size and τ is the generation time). The thick solid line indicates the median estimates and the shaded area indicates the 95% highest posterior density

No recombination events were detected in the VP1 or 3D^pol^ region. High support values (defined as PP >0.9) were obtained for four genotypes in the VP1 tree (Genotypes II–V; GII–GV) and for five genotypes in the 3D^p^°^l^ tree (Genotypes A–E; GA–GE) (Figs. 1 and 2). Both the VP1 and 3D^pol^ trees contained spatiotemporally structured clusters, but the VP1 and 3D^pol^ trees were clearly incongruent. Based on a genetic discrimination rate of at least 15%, five clusters were obtained in the VP1 region (Fig. 1). In the ascent cluster (cluster 1), the prototype Ohio strain (isolated in the US in 1947) and Taiwan strain 01 (isolated in 1988) were clustered together in VP1 and in 3D^pol^ (GA). However, the support value was not statistically significant for VP1. In both the VP1 and partial 3D^pol^ regions, Taiwan strain 01 had the same sequence as the prototype Ohio strain. Strains GII (1994–2013), GIV (1999–2003), and GV (2007–2009) were isolated in Asia, where GIV was geographically distributed in India. By contrast, GIII was isolated in France (2006–2010) and Australia (2005). The analysis of the historical transmission routes for VP1 by using the Spread program showed that only the Taiwan-South Korea route had a BF of 6.67. The demographic history determined by BSP showed that the CV-B2 viral population had maintained a stable level with only a slight decrease since 1947, where the median Neτ was 3.57–3.42 (Fig. 3a). Compared with the data reported in year 2000, the Neτ for CV-B2 was much lower than the median Ne*τ* values of 4 × 10^3^ reported for EV68 [33] and 30 for CV-B5 [34]*.*

Phylogenetic comparisons of the VP1 topology with those of other EVs revealed similar features: (1) a serotype-based monophyletic origin; (2) different CV-B2 genotypes evolved and diverged sequentially, where each genotype had a different geographic distribution and circulation half-life; (3) the prevalent lineages were chronologically replaced by emerging different lineages that co-existed at the same time and in the same location and (4) tree topologies with long internal branches (i.e., the branch from the node near the root) and intricate short terminal branches [35]. In particular, the long internal branches and short terminal branches indicated a stable pathogen population size [36], which supported BSP estimation of the CV-B2 viral population in this study.

Five genotypes are depicted in the 3D^pol^ tree (Fig. 1b). The ancestral America strains (1947–1996) were clustered in GA–C, especially in GB. By contrast, most of the Taiwan strains were clustered in GD–GE with the other Asian strains. Interestingly, GB in the 3D^pol^ tree included two Taiwan strains isolated during 2011–2013 (clustered in the VP1 region of GV). This indicated that those strains with GV sequence in VP1 region has endemic in Taiwan since 2002–2013, but the 3D^pol^ gene might have switched from GE to GB, because the 3D^pol^ gene in the latter two isolates (2011 and 2013) clustered together with US strains in GB. Therefore, changes in the trends in CV-B2 in Taiwan should be monitored continuously in Taiwan.

In contrast to the low (< 4) median Neτ value estimated by VP1 region of CV-B2 in this study, the demographic history analysis of the 3D^pol^ region by BSP showed that CV-B2 emerged during 1947, where it had a larger Neτ value of 27.8 before it began to bottleneck in 1955. After peaking in 1985 the Neτ values decreased gradually from 80.4 to 51.7 during 1985–2013 (Fig. 3b). Only capsid-based analyses reveal that serotypes of EVs are monophyletic; however, when the analysis is based on regions outside the capsid region, EVs are monophyletic species rather than serotypes [37]. Thus, the 3D^pol^-based estimation of Neτ was relevant to EV-B species (CV-B2 is one of the EV-B serotypes). One proposed explanation for this phenomenon is independent evolution of VP1 and 3D^pol^ [38], which is consistent with the discordant topologies of VP1 and 3D^pol^ determined in the present study. Our results indicate that recombination may occur within discordant strains, and that mutation in the capsid region is a result of immune escape.

However, this study detected high (>95 %) aa sequence similarity. Based on the *p*-distances calculated in this study, the estimated maximum differences in the 846-nt (2456–3301) and 282-aa sequences of the VP1 gene were 80.0 % and 96.8 %, respectively. The estimated maximum differences in the 441-nt (6641–7087) and 147-aa sequences of the partial 3D^pol^ gene were 74.7 % and 91.9 %, respectively. Further, the estimated evolutionary rates of VP1 and 3D^pol^ were 5.42 × 10^−3^ (95 % HPD: 3.38 × 10^−3^–6.98 × 10^−3^) and 6.02 × 10^−3^ (3.72 × 10^−3^–8.31 × 10^−3^) substitutions/site/year (s/s/y), respectively. The VP1 mutation rate of CV-B2 is within the mutation rate range reported in a previous study of EVs (range from 3.40 × 10^−3^ to 1.19 × 10^−2^ s/s/y) [39]. In the present study, the estimated evolution rate of 3D^pol^ (6.02 × 10^−3^ s/s/y) was not only higher than that of VP1 but also higher than the estimated evolution rate reported for 3D^pol^ in previous studies of EVs (from 5.53 × 10^−3^ to 1.17 × 10^−2^ ns/s/y) [39]. The d*N*/d*S* values for the VP1 and 3D^pol^ region were 0.0334 and 0.0265, respectively, but neither region contained a positive selection site. In summary, the low and slightly decreasing Neτ value, low evolution rate, and negative selection indicate that the variation in this VP1 region was fixed by adaptive fitness.

Phylodynamic analysis depicts interaction among epidemics, evolution and selection and reveals the demographic and spatiotemporal transmission signatures [26]. A phylogenetic tree with an imbalanced trunk and short terminal branches suggests acute infection with partial cross-immunity, for example, the phylogenetic tree topology for seasonal influenza virus is ladder-like. In contrast, the topology of a chronic infection such as hepatitis C virus is star-like with long terminal branches [26]. The tree balance is impacted by selection; therefore, a ladder-like backbone indicates the trajectory of fitness. Table 2 summarizes the results of the statistical analyses of tree shape and balance obtained in the present study. Fu and Li’s *D* value is a classic summary statistic for testing neutrality, which indicates the normalize difference between the external branch lengths and total tree length. As the symmetry of a phylogeny increases, the values for *B*_*1*_ and *Cn* are expected to increase, whereas the value of *Ic* is expected to decrease. The distributions of the mean *Ic* indices among human and zoonotic RNA viruses reportedly peak between 0.15 and 0.2 [32]. The values for the VP1 tree were within this range in the present study, but 3D^pol^ had higher tree balance indices compared with VP1.

**Table 2:** Summary statistics used in tests of demographic neutrality and tree balance

Gene region	Statistical measures	Mean	Median	95 % HPD^a^
VP1	*D* ^b^	-2.9149	-2.933	-3.5863, -2.1837
	*B*_*1*_^c^	24.9094	24.9591	22.9639, 26.9044
	*Cn* ^d^	15.7291	16	14, 18
	*Ic* ^d^	0.1969	0.1902	0.1682, 0.2384
3D	*D*	-2.1294	-2.1457	-2.818, -1.4114
	*B*_*1*_	16.7549	16.6385	15.5468, 18.0199
	*Cn*	11.0685	11	10, 12
	*Ic*	0.238	0.2386	0.1875, 0.2822

^a^HPD Highest posterior density

^b^ *D* Fu and Li’s D: Normalized difference between the external branch lengths and total tree length. This is a classic summary statistic used for testing neutrality

^c^ *B*_*1*_ Maximum number of nodes between an internal node and the tips of the tree. A larger value indicates a more balanced tree

^d^ *Cn* Cherry count, the number of internal nodes with exactly two terminal branches. A larger number indicates a more balanced tree

^e^ *Ic* Colless’s tree imbalance index with a range of [0, 1]. A larger number indicates a more imbalanced tree

A major challenge in modern phylodynamics is qualitatively and quantitatively describing a tree topology to elucidate the epidemiological, evolutionary, and demographic characteristics of a pathogen. To investigate the evolutionary history of an endemic or sporadic pathogen, a BMCMC sampling framework was used to infer time scaled phylogenies of CV-B2 during 1947–2013. Sampling bias was unavoidable in this study because the incomplete GenBank data precluded sampling of all strains isolated in earlier time periods (particularly before the 1980s). An even greater problem was that the sequences tended to accumulate in the same isolation years and locations because CV-B2 outbreaks are rare. On average, CVB2 has been implicated in 1.5 %–6.0 % of the reported surveillance results for known EV serotypes, and thus the low frequency of CV-B2 outbreaks may be related to the stable low Ne*τ* values estimates obtained in this study. Furthermore, low herd immunity selection may have an important role in endemic EVs because the VP1 has high aa sequence similarity and a low rate of evolution. Compare to 3D^pol^ region, the VP1 region has a more balanced topology.

## Conclusions

An endemic or sporadic EV, CV-B2, was analyzed in a phylodynamic analysis, and four genotypes in the VP1 region and five genotypes in the 3D^pol^ region were determined. Recombination events were not detected in the VP1 sequences or 3D^pol^ sequences, but VP1 and 3D^pol^ trees were clearly incongruent, thereby indicating the occurrence of recombination events involving the region between VP1 and 3D^pol^. Although the random branching topology observed in this study is typically interpreted as the rapid evolution of an RNA virus, the imbalanced tree topology and the high similarity of the VP1 sequences indicate that endemic CV-B2 is characterized by a low viral population and low herd immune selection.

### Availability of supporting data

See additional files for supporting data.

## Additional files

Additional file 1:
**Annual reported rate of coxsackievirus B2 (CV-B2) in Taiwan.** (TIFF 686 kb) Additional file 2:
**Global surveillance of coxsackievirus B2.** (PDF 6 kb) Additional file 3:
**List of sampled coxsackievirus B2 (CV-B2) strains.** (PDF 14 kb) Additional file 4:
**Models compared by an extension of air carrier investment model (ACIM).** (PDF 10 kb)

## Abbreviations

aa: Amino acid
BF: Bayes factor
BEAST: The Bayesian Evolutionary Analysis Sampling Trees
BMCMC: Bayesian Monte Carlo Markov Chain
BSP: Bayesian skyline plot
*Cn*: Effective sample size
CV: Coxsackievirus
EV: Enterovirus
ESS: Effective sample size
GTR: Generalised Time Reversible
HPD: Highest probability density
*Ic*: Colless’s tree imbalance
MCC: Maximum clade credibility
Neτ: Effective population size over time
nt: Nucleotide
PP: Posterior probability
SLAC: Single Likelihood Ancestor Counting
SRD06: Shapiro-Rambaut-Drummond-2006
TMRCA: Time to most recent common ancestor
uced: Uncorrelated exponential distribution
