# Supplementary figures and images for: Phylodynamic reconstruction of the spatiotemporal transmission and demographic history of coxsackievirus B2
Source: BMC Bioinformatics. 2015 Sep 21;16:302. doi: 10.1186/s12859-015-0738-2 (PMC4578604; doi:10.1186/s12859-015-0738-2)

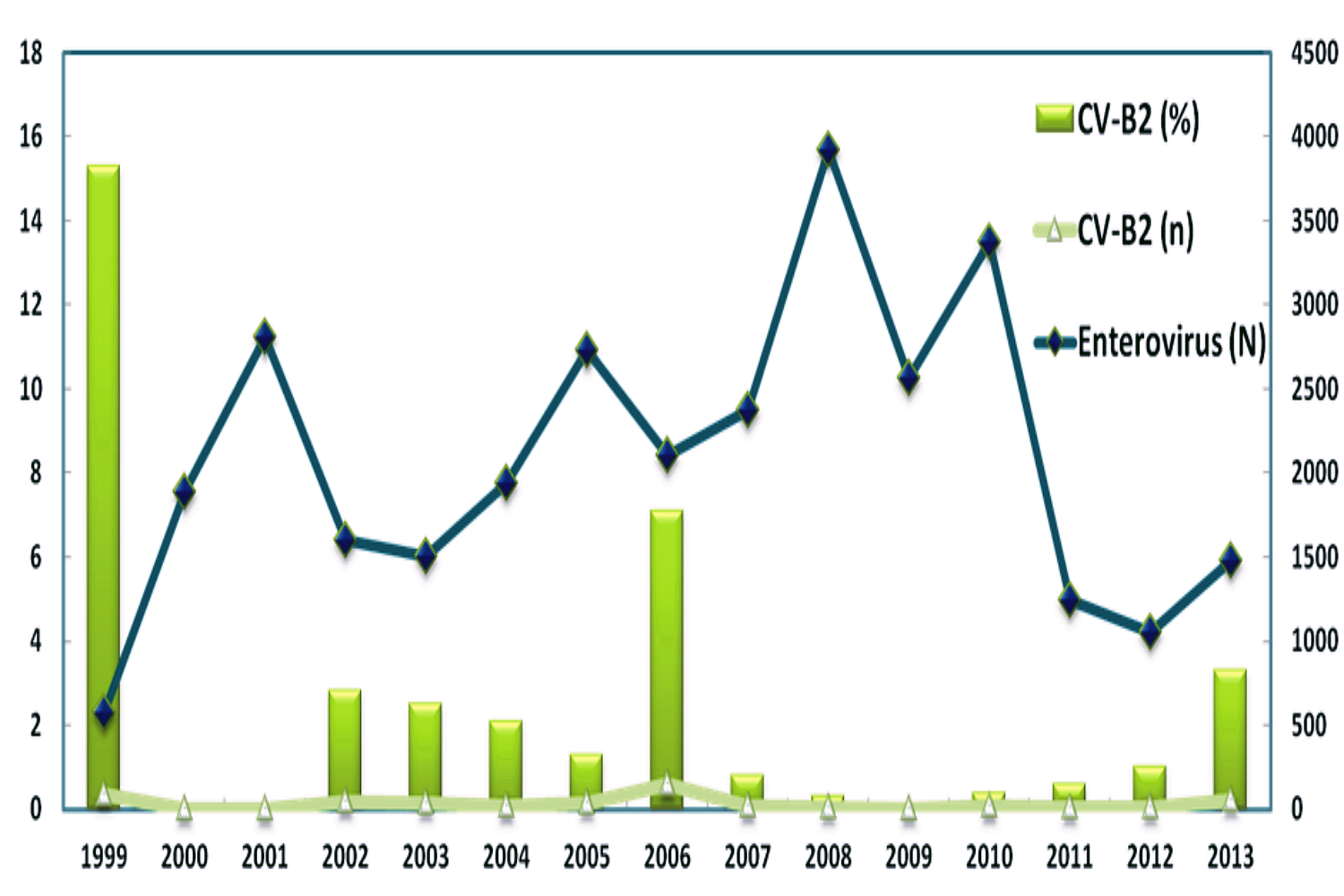

Supplement: Additional file 1: — Annual reported rate of coxsackievirus B2 (CV-B2) in Taiwan. (TIFF 686 kb) [file 12859_2015_738_MOESM1_ESM.tiff]
