# Supplementary material for: Phylodynamic reconstruction of the spatiotemporal transmission and demographic history of coxsackievirus B2
Source: BMC Bioinformatics. 2015 Sep 21;16:302. doi: 10.1186/s12859-015-0738-2 (PMC4578604; doi:10.1186/s12859-015-0738-2)
Supplement: Additional file 2: — Global surveillance of coxsackievirus B2. (PDF 6 kb) [file 12859_2015_738_MOESM2_ESM.pdf]

Additional file 2 – Global surveillance of coxsackievirus B2

Country	Year	Annual reported rate % (cases)	Reference
Taiwan	1999-2012	1.5 (444)	From CDC-TW
South Korea	1999-2011	6.0 (187)	[3]
Cyprus	2003-2007	4.3 (6)	[4]
France	2000-2004	2.9 (81)	[5]
Germany	2000-2005	2.0 (14)	[6]
Tunisia	1992-2003	2.1 (5)	[7]
United States	1970-2005	5.2 (36)	[1]
