# Supplementary material for: Phylodynamic reconstruction of the spatiotemporal transmission and demographic history of coxsackievirus B2
Source: BMC Bioinformatics. 2015 Sep 21;16:302. doi: 10.1186/s12859-015-0738-2 (PMC4578604; doi:10.1186/s12859-015-0738-2)
Supplement: Additional file 3: — List of sampled coxsackievirus B2 (CV-B2) strains. (PDF 14 kb) [file 12859_2015_738_MOESM3_ESM.pdf]

Additional file 3 – List of sampled coxsackievirus B2 (CV-B2) strains.

Accession number		Strain name	Isolation Year	Isolation location				
VP1	3D			Symbol	Country	City	Latitude	Longitude
AB862097	LC057297	1	1988	TW	Taiwan		23.011	120.666
AB862101	LC057298	5	1999	TW	Taiwan		23.011	120.666
AB862107	LC057299	11	2005	TW	Taiwan		23.011	120.666
AB862115	LC057300	19	2010	TW	Taiwan		23.011	120.666
AB862116	LC057301	20	2011	TW	Taiwan		23.011	120.666
LC055763	LC057302	25	2002	TW	Taiwan		23.011	120.666
LC055764	LC057303	26	2002	TW	Taiwan		23.011	120.666
LC055765	LC057304	27	2003	TW	Taiwan		23.011	120.666
LC055766	LC057305	28	2003	TW	Taiwan		23.011	120.666
LC055768	LC057306	30	2003	TW	Taiwan		23.011	120.666
LC055769	LC057307	31	2003	TW	Taiwan		23.011	120.666
LC055770	LC057308	32	2003	TW	Taiwan		23.011	120.666
LC055771	LC057309	33	2004	TW	Taiwan		23.011	120.666
LC055772	LC057310	34	2006	TW	Taiwan		23.011	120.666
LC055773	LC057311	35	2006	TW	Taiwan		23.011	120.666
LC055774	LC057312	36	2006	TW	Taiwan		23.011	120.666
LC055775	LC057313	37	2006	TW	Taiwan		23.011	120.666
LC055776	LC057314	38	2007	TW	Taiwan		23.011	120.666
LC055777	LC057315	39	2013	TW	Taiwan		23.011	120.666
LC055778	LC057316	40	2008	TW	Taiwan		23.011	120.666
EF174468	EF174468	243	2004	KR	South Korea		35.908	127.767
EF174469	EF174469	279	2004	KR	South Korea		35.908	127.767
AF085363	AF085363	Ohio	1947	USoh	United States	Ohio	40.417	-82.907
KM386639	KM386639	BCH314	2007	CNbj	China	Beijing	39.9025	116.427
AF225468		2	1987	CNyn	China	Yunnan	25.045	102.71
GQ329742		147	1994	CNsd	China	Shandong	36.669	117.02
GQ329743		151	1997	CNsd	China	Shandong	36.669	117.02
GQ329738		163	2000	CNsd	China	Shandong	36.669	117.02
GQ329739		233	2000	CNsd	China	Shandong	36.669	117.02
GQ329741		363	2004	CNsd	China	Shandong	36.669	117.02
GQ329740		425	2003	CNsd	China	Shandong	36.669	117.02
AY373212		10169	1976	USnd	United States	North Dakota	47.551	-101
AY373213		10170	1977	USne	United States	Nebraska	41.493	-99.902
AY373214		10171	1971	USma	United States	Mississippi	32.3547	-89.399
AY373216		10173	1980	USal	United States	Mississippi	32.3182	-86.902
AY373217		10174	1981	USma	United States	Mississippi	32.3547	-89.399
AY373218		10175	1982	USma	United States	Mississippi	32.3547	-89.399
AY373219		10176	1983	USma	United States	Mississippi	32.3547	-89.399
AY373220		10177	1991	USwa	United States	Washington	38.907	-77.036
AY373222		10179	1992	USfl	United States	Florida	27.665	-81.516

Accession number		Strain name	Isolation	Isolation location				
VP1	3D		Year	Symbol	Country	City	Latitude	Longitude
AY373223		10180	1996	AR	Argentina		-38.416	-63.617
HG793667		175013	2010	FRcf	France	Clermont-Ferrand	45.777	3.087
AM711076		345042	2006	FRcf	France	Clermont-Ferrand	45.777	3.087
FJ868285		2041577	2005	AUsy	Australia	Sydney	-33.867	151.207
HF948083		CF07508	2007	FRcf	France	Clermont-Ferrand	45.777	3.087
AM711056		CSF200065	2006	FRcf	France	Clermont-Ferrand	45.777	3.087
KC867081		JB141090057	2009	CNsz	China	shenzhen	23.132	113.267
KC867082		JB141230200	2012	CNsz	China	shenzhen	23.132	113.267
JN655889		M10MG17	2010	CNjg	China	Jiangsu	32.456	119.923
JX513570		M81	2008	INka	India	Karnataka	15.317	75.714
HQ454497		N1	2007	INka	India	Karnataka	15.317	75.714
JN203579		N1002	2009	INup	India	Uttar Pradesh	27.571	80.098
JN203580		N1054	2009	INup	India	Uttar Pradesh	27.571	80.098
JN203573		N430	2008	INup	India	Uttar Pradesh	27.571	80.098
JN203574		N454	2008	INup	India	Uttar Pradesh	27.571	80.098
JN203568		N68	2007	INka	India	Karnataka	15.317	75.714
JN203569		N69	2007	INka	India	Karnataka	15.317	75.714
JN203576		N791	2009	INke	India	Kerala	10.851	76.271
JN203577		N797	2009	INup	India	Uttar Pradesh	27.571	80.098
JN203578		N816	2009	INup	India	Uttar Pradesh	27.571	80.098
JN203571		N82	2007	INka	India	Karnataka	15.317	75.714
