# Supplementary material for: Phylodynamic reconstruction of the spatiotemporal transmission and demographic history of coxsackievirus B2
Source: BMC Bioinformatics. 2015 Sep 21;16:302. doi: 10.1186/s12859-015-0738-2 (PMC4578604; doi:10.1186/s12859-015-0738-2)
Supplement: Additional file 4: — Models compared by an extension of air carrier investment model (ACIM). (PDF 10 kb) [file 12859_2015_738_MOESM4_ESM.pdf]

Additional file 4 – Models compared by an extension of air carrier investment model (ACIM)

(A) VP1

Trace[a]	AICM[b]	S.E.[c]	SRD UCED CON	SRD UCED BSP	SRD UCLD CON	SRD UCLD BSP	GTRG UCLD CON	GTRG UCED BSP	GTRG UCLD CON	GTRG LOG BSP
SRD UCED CON	12668.652*	+/- 0.147	-	6.222	35.833	35.411	645.636	645.28	673.689	680.538
SRD UCED BSP	12674.874	+/- 0.115	-6.222	-	29.611	29.189	639.414	639.058	667.467	674.316
SRD UCLD CON	12704.485	+/- 0.109	-35.833	-29.611	-	-0.422	609.803	609.447	637.856	644.705
SRD UCLD BSP	12704.063	+/- 0.137	-35.411	-29.189	0.422	-	610.225	609.869	638.278	645.127
GTRG UCLD CON	13314.288	+/- 0.134	-645.636	-639.414	-609.803	-610.225	-	-0.356	28.053	34.902
GTRG UCLD BSP	13313.932	+/- 0.09	-645.28	-639.058	-609.447	-609.869	0.356	-	28.409	35.258
GTRG LOG CON	13342.341	+/- 0.167	-673.689	-667.467	-637.856	-638.278	-28.053	-28.409	-	6.849
GTRG LOG BSP	13349.190	+/- 0.146	-680.538	-674.316	-644.705	-645.127	-34.902	-35.258	-6.849	-

(B) 3D

Trace	AICM	S.E.	SRD UCED CON	SRD UCED GSP	SRD UCLD CON
SRD UCED CON	6992.433*	+/- 0.064	-	2.592	17.48
SRD UCED GSP	6995.026	+/- 0.113	-2.592	-	14.887
SRD UCLD CON	7009.913	+/- 0.158	-17.48	-14.887	-

a. Two substitution models were used: a Generalised Time Reversible model with Gamma distribution (GTRG) and a Shapiro-Rambaut-Drummond-2006 (SRD) model; two molecular clock models were used: an uncorrelated lognormal distribution (UCLD) relaxed clock model and an uncorrelated exponential distribution (UCED) relaxed clock model. For pairwise comparisons, each clock model was combined with the following four demographic history models: constant size (CON) and Bayesian skyline plot (BSP). Only the effective sample sizes of major estimated parameters >200 were compared.

b. To identify the model combination with the best fit to the sequence data analyzed, the AICM was estimated using Tracer 1.6 program. Low AICM values were indicated better interpreted as a good model fit. Differences in AICM estimates are reported. A positive value indicates a better relative model fit of the model in the row compared to the model in the column. The best model is indicated with an asterisk (*).

c. Standard error for the marginal likelihood.
